# Lymphoedema management in podoconiosis

**DOI:** 10.1016/S2214-109X(18)30330-9

**Published:** 2018-09-01

**Authors:** Meseret Molla, Moses Ngari, James A Berkley, Patricia Njuguna, Greg Fegan, Trudie Lang, Melanie J Newport, Fikre Enquoselassie, Gail Davey

**Affiliations:** Centre for Environmental and Developmental Studies; KEMRI Wellcome Trust Research Programme, Kilifi, Kenya; KEMRI Wellcome Trust Research Programme, Kilifi, Kenya; Centre for Tropical Medicine and Global Health, University of Oxford, Oxford, UK; KEMRI Wellcome Trust Research Programme, Kilifi, Kenya; KEMRI Wellcome Trust Research Programme, Kilifi, Kenya; Swansea University Medical School, Swansea, UK; KEMRI Wellcome Trust Research Programme, Kilifi, Kenya; Centre for Tropical Medicine and Global Health, University of Oxford, Oxford, UK; Addis Ababa University, Addis Ababa, Ethiopia; Wellcome Trust Centre for Global Health Research, Brighton and Sussex Medical School, Brighton, BN1 9PX, UK; School of Public Health; School of Public Health; Addis Ababa University, Addis Ababa, Ethiopia; Wellcome Trust Centre for Global Health Research, Brighton and Sussex Medical School, Brighton, BN1 9PX, UK

## Authors reply

We thank Jill Brooks and colleagues for their comments on how their trial was referred to in our article.^1^

We used the adjective “small” to provide context for readers of *The Lancet Global Health.* Given that the average number of individuals in the nine non-stepped-wedge trials reported so far in this journal in 2018 was over 3600, both the Brooks trial^2^ and GoLBeT^1^ are small by comparison. Small trials can be highly efficient, and the term is not used pejoratively.

The point we were trying to make in the introduction was around the type of question GoLBeT was designed to answer, and how this differed from earlier studies. One earlier study was uncontrolled,^3^ while the Brooks and colleagues^2^ trial compared different approaches to foot hygiene, rather than foot hygiene versus no foot hygiene. Brooks and colleagues^2^ showed less transepidermal water loss and greater stratum corneum hydration with glycerol than with water alone, whereas GoLBeT tested a composite package versus no package. The question GoLBeT was designed to answer came from a public health perspective within the Ethiopian Federal Ministry of Health, who asked for evidence of the effectiveness of the most basic package of treatment, compared with none, under pragmatic field circumstances. This research aimed to assist decisions on the value, or absence of value, of scale-up of provision of lymphoedema care through the state health system of Ethiopia.

Lastly, we recognise that Brooks and colleagues investigated days of work lost to acute adenolymphangitis, a secondary outcome of GoLBeT, which differs from acute dermatolymphangioadenitis incidence. We applaud the Brooks team for measuring transepidermal water loss and stratum corneum hydration, which we agree are important in the pathogenesis of podoconiosis and its sequelae. Our primary outcome was selected from a public health perspective, following consultation with patients, carers, and service providers who identified acute dermatolymphangioadenitis incidence as the most debilitating consequence of the disease.
